# Establishing and evaluating physician-pharmacist collaborative clinics to manage patients with type 2 diabetes in primary hospitals in Hunan province: study protocol of a multi-site randomized controlled trial in the era of COVID-19 pandemic

**DOI:** 10.1186/s12913-022-07653-8

**Published:** 2022-03-04

**Authors:** Sheng-Lan Tan, Jie Xiao, Hai-Yan Yuan, Lei Chen, Qing Wang, Da-Xiong Xiang, Xia Li, Yan-Gang Zhou, Yan Guo, Hai-Ying Huang, Dan-Hui Zhao, Yue Li, Li Wang, Qun Li, Juan Liu, Ping Xu

**Affiliations:** 1 Department of Pharmacy, The Second Xiangya Hospital, Central South University, Changsha, 410011 China; 2 Institute of Clinical Pharmacy, The Second Xiangya Hospital, Central South University, Changsha, 410011 China; 3 Department of Endocrine, The Second Xiangya Hospital, Central South University, Changsha, 410011 China; 4 Department of Pharmacy, Taoyuan People’s Hospital, Changde, 415700 China; 5 Department of Pharmacy, The People’s Hospital of Liuyang, Changsha, 410300 China; 6 Department of Pharmacy, The First People’s Hospital of Pingjiang, Yueyang, 410400 China; 7 Department of Pharmacy, People’s Hospital of Ningxiang, Changsha, 410600 China; 8 Department of Pharmacy, Yueyang Central Hospital, Yueyang, 414020 China; 9 Department of Pharmacy, The Second People’s Hospital of Huaihua, Huaihua, 418000 China; 10 Department of Pharmacy, Yiyang Central Hospital, Yiyang, 413000 China

**Keywords:** Diabetes, Pharmacist, Collaborative clinics, Rural area, RCT

## Abstract

**Background:**

The COVID-19 pandemic has exerted an unprecedented and universal impact on global health system, resulting in noticeable challenges in traditional chronic disease care, of which diabetes was reported to be most influenced by the reduction in healthcare resources in the pandemic. China has the world’s largest diabetes population, and current diabetes management in China is unsatisfactory, particularly in rural areas. Studies in developed countries have demonstrated that physician-pharmacist collaborative clinics are efficient and cost-effective for diabetes management, but little is known if this mode could be adapted in primary hospitals in China. The aim of this proposed study is to develop and evaluate physician-pharmacist collaborative clinics to manage type 2 diabetes mellitus (T2DM) in primary hospitals in Hunan province.

**Methods:**

A multi-site randomized controlled trial will be conducted to evaluate the effectiveness and cost-effectiveness of the physician-pharmacist collaborative clinics compared with usual care for Chinese patients with T2DM. Six primary hospitals will participate in the study, which will recruit 600 eligible patients. Patients in the intervention group will receive services from both physicians and pharmacists in the collaborative clinics, while the control group will receive usual care from physicians. Patients will be followed up at the 3rd, 6th, 9th and 12th month. Comparison between the two groups will be conducted by assessing the clinical parameters, process indicators and costs on diabetes. A satisfaction survey will also be carried out at the end of the study.

**Discussion:**

If effective, the physician-pharmacist collaborative clinics can be adapted and used in primary hospitals of China to improve glycemic control, enhance medication adherence, decrease incidence of complications and reduce patients’ dependence on physicians. Findings from the present study are meaningful for developing evidence-based diabetes care policy in rural China, especially in the COVID-19 pandemic era.

**Trial registration:**

Chinese Clinical Trial Registry, ChiCTR2000031839, Registered 12 April 2020.

## Background

Globally, as of January 21, 2022, the COVID-19 pandemic has infected over 340.54 million confirmed cases and killed more than 5.57 million people worldwide, reported to WHO [1]. Currently, the Omicron variant has been detected worldwide and will spread more easily than the original SARS-CoV-2 virus, which will increase the number of new COVID-19 cases exponentially. Experts postulated that this pandemic may persist for the next few years and may even recur seasonally.

The COVID-19 pandemic has resulted in an unprecedented global health crisis, challenging routine health systems’ ability to cope with the pandemic and disrupting current best practices for other disease management [2, 3]. In order to cope with the pandemic, countries across the world have transferred a large number of physicians and nurses to treat the confirmed cases, conduct nucleic acid tests and make COVID-19 vaccine shots, resulting in shortage of medical resources for other diseases treatment. Thus, many countries have to limit standard outpatient clinics and decrease inpatient capacity. The COVID-19 pandemic has placed a noticeable effect on patients with chronic diseases who require long-term care, of which diabetes was reported to be most influenced by the reduction in healthcare resources in the pandemic [4]. Besides the difficulties of shortage in medical staff and standard outpatient clinics, delayed care seeking, limited self-care practice, transportation inconveniency and unidentified adverse events all contributed to the major challenges of traditional diabetes care in the COVID-19 era [5].

With high-speed economic growth and lifestyle changes in the last two decades, China currently has the world’s largest diabetes population, which is still growing rapidly. The prevalence of diabetes in China was reported to be 0.67% in 1980 and increased to 11.9% in the latest published nationwide epidemic investigation in 2021 [6]. Diabetes has become a major cause of mortality in China, and about 1.27 million deaths was reported attributable to diabetes annually according to the estimates from IDF (International Diabetes Federation) Diabetes Atlas in 2013 [7]. Similar to other countries, the majority of patients with diabetes in China are type 2 diabetes mellitus (T2DM). The direct medical costs of type 2 diabetes and its complications in China were estimated to be 26.0 billion USD in 2007 and to be 47.2 billion USD in 2030 [8].

So far, the predominant care mode of diabetes management in China is the traditional physician-led mode. Physicians are responsible to diagnose, treat and educate patients. Present diabetes management in China is unsatisfactory, particularly in rural areas. As reported in a national investigation, patients with T2DM in urban areas of China, 43.1% were aware of their diagnosis, 38.4% were treated, and only 53.3% of the treated patients had adequate glycemic control. The rates in the rural regions were even lower, which were merely 29.1%, 25.2% and 42.3%, respectively [6].

In developed countries, numerous pharmacists have expanded their roles from medication dispensers to integrated members of interdisciplinary healthcare teams by providing the service of medication therapy management (MTM), which is defined as “a service or group of services that optimize therapeutic outcomes for individual patients” [9]. Those pharmacists, with their pharmacotherapeutic knowledge and patient centered training, are qualified and irreplaceable partners of physicians to educate and monitor patients on medication therapies, which ultimately improve disease management, medication adherence and overall health-related quality of life [10, 11]. Based on a comprehensive literature review, we found that diabetes management participated by pharmacists can significantly enhance glycemic control, improve concomitant disease management, delay the risks for complications and is cost-effective compared to usual care [12–17]. Those studies suggest that the mode of a physician-pharmacist collaborative clinic may be an alternative effective strategy to manage T2DM in China.

So far, the physician-pharmacist collaborative clinic is gaining recognition in some tertiary hospitals in China. However, to our knowledge, no collaborative clinics have been set up in primary hospitals in China yet, and one major reason is probably that the majority of pharmacists in primary hospitals are not confident or qualified to provide MTM services. According to China's hierarchical health care system, patients in primary hospitals usually have mild conditions with a few complications or comorbidities, and those with severe conditions are usually treated in tertiary hospitals. Therefore, if pharmacists could provide MTM services at the early stage of diabetes in primary hospitals in China, it may be very effective and promising to control and delay the progress of diabetes. However, little is known about the efficacy and cost-effectiveness of physician-pharmacist collaborative clinics for T2DM management in primary hospitals in China.

Therefore, we proposed to establish physician-pharmacist collaborative clinics to manage T2DM in primary hospitals in China. Theoretically, the physician-pharmacist collaborative clinics could alleviate the shortage of medical staff, provide patient education in clinics, reduce patients’ burden of frequent follow ups in tertiary hospitals and decrease patients’ dependence on physicians in primary hospitals, which is especially important in the era of COVID-19 pandemic. This paper reports the development and study procedure of establishing and evaluating physician-pharmacist collaborative clinics to manage T2DM in primary hospitals in Hunan province.

### Aim

This study aims to improve patient’s glucose control and medication adherence, reduce hospitalization or emergency room visits and incidence of complications related to diabetes, and decrease costs on diabetes by implementing physician-pharmacist collaborative clinics in primary hospitals during the COVID-19 pandemic. Moreover, our study will provide experiences to expand the mode of physician-pharmacist collaborative clinics in primary hospitals across China to manage other chronic diseases during and after the COVID-19 pandemic.

## Methods/design

### Study design

**Fig. 1:**
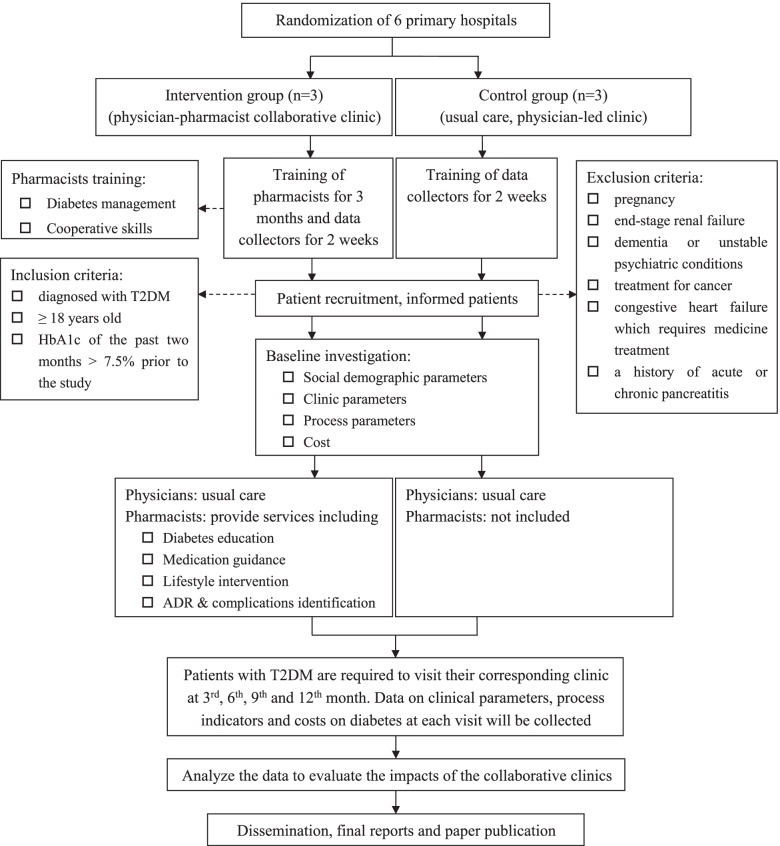
Flowchart of the study

The study is a multi-site randomized controlled trial. We will carry out the study in six primary hospitals in Hunan province. Eligible patients will be randomly assigned to the intervention group (physician-pharmacist collaborative clinics) or the control group (usual care clinics led by a physician) and will be followed up at the 3rd, 6th, 9th and 12th month. Comparison between the two groups will be conducted by assessing the clinical parameters, process indicators and costs on diabetes. Fig. 1 describes the study design.

### Study participants and inclusion criteria

Patients eligible for this project have to comply with all of the requirements below: have been diagnosed with T2DM; male or female, over the age of 18 years old; the HbA1c level of the past two months was > 7.5% prior to the study and an informed consent signed by the patient or a legal guardian. Patients will be excluded if: pregnancy; have end-stage renal failure (hemodialysis or glomerular filtration rate < 10 mL/min); have dementia or unstable psychiatric conditions; receive treatment for cancer; have congestive heart failure which requires medicine treatment or have a history of acute or chronic pancreatitis. All eligible patients who agree to participate the study have to sign the informed consent form before being enrolled.

Pharmacists eligible for this project have to comply with all of the requirements below: have worked as a clinical pharmacist at the selected primary hospital for over 2 years, with priority given to those with diabetes management experience; have received a standardized training as a clinical pharmacist before this project, with priority given to those in endocrinology specialty; be able to get comprehensive training in the Second Xiangya Hospital for three months and can complete the project.

### Study settings

**Fig. 2:**
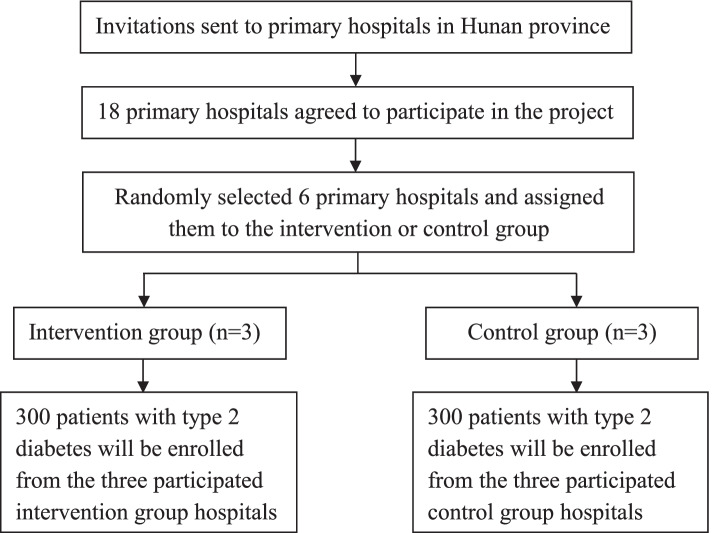
Flow-chart of setting selection and number of patients in the project

Currently in Hunan province, county hospitals, township health centers, and village clinics are the major primary health care providers in rural areas. As there are limited pharmacists and their main responsibility is dispensing in village clinics and township health centers, we chose county hospitals as the research sites. Since there are several county hospitals in each county, we selected the best one in that county as our studied primary hospital with the aim of homogenizing the quality of the settings. We divided these counties into three subgroups based on their per capita income levels, namely high, medium and low incomes counties. We sent invitation letters to 27 primary hospitals and 18 agreed to participated in the study. Finally, we randomly selected six of them (two in each subgroup) to conduct the study as shown in Fig. 2.

### Procedures

#### Training pharmacists and data collectors

Three pharmacists from the primary hospitals assigned to the intervention group will be trained for three months in the Second Xiangya Hospital. The training courses mainly include two aspects, namely diabetes management and cooperative skills, as shown in Table 1. These pharmacists are required to spend most of their training time either in the outpatient clinic of endocrinology or in the wards of endocrinology to get as much experience as possible. Besides the traditional teaching methods, problem-based learning and scenario simulation learning methods will be implemented to train the pharmacists. Serial tests will be carried out to examine how well the knowledge and skills they have mastered.

**Table 1:** Training courses for pharmacists from primary hospitals

Training courses	Details	time
Diabetes management	1.Theoretical knowledge □ Basic knowledge of diabetes, including identification of diabetes complications □ Basic knowledge of hypoglycemic a gents(oral & injection medicine) □ treatment target of blood glucose level □ Laboratory tests interpretation □ Medication adherence □ Diet □ Exercise □ lifestyle 2. Practice and Application in patients 3. Six tests to assess their theoretical knowledge and practice skills	Two months
Cooperative skills	1. Theoretical knowledge on communication skills 2. Practice in outpatient clinics and inpatient wards	One month

Six nurses from each of the primary hospitals will be enrolled as data collectors and will be trained for two weeks in the Second Xiangya Hospital. They will be simply introduced about the background of the project, namely diabetes management and pharmacotherapy. These data collectors will get detailed training of patient enrollment talk, informed consent signature and data collection for assessments. Data collectors will be trained to finish both the printed and the electronic CRF (case report form) forms for assessments.

#### Recruitment and withdrawn

At the stage of patient enrollment in each clinic, a trained data collector will stay in the clinic and prepare to talk to patients. Once the physician identifies a potential eligible patient with T2DM in the clinic and simply introduces the project to him/her, the trained data collector will explain the project in details. If the patient agrees to join in the project, the trained data collector will ask the patient to sign the informed consent, and help him/her to fill out the printed CRF form as baseline assessment.

Patients enrolled will be withdrawn from the project if any of the conditions occurs: the patient decides not to continue in the project for any reason; the patient is lost for follow-up for at least twice; the researcher considers the patient is no longer physically and/or psychologically fit to remain in the study. The reasons for withdrawn will be recorded.

#### Baseline interview

Baseline investigations will be carried out in all the participated six primary hospitals. The contents of the investigations include four aspects, namely socio-demographic parameters, clinical parameters, process indicators and costs on diabetes as shown in Table 2. Data collectors are responsible to guide enrolled patients to finish the baseline investigations. The information of body weight, height, waistline and blood pressure will be measured and recorded in the clinic by the data collector. The Glucose and lipid files will be measured by lab tests. Data of patient socio-demographic parameters, process indicators and costs on diabetes will be provided by the patients or their guardians.

**Table 2:** Services to be provided by pharmacists in the primary hospitals

Pharmacists’ services	Details	Methods
Diabetes education	□ Disease introduction □ Control target of blood glucose □ The types and principles of hypoglycemic agents □ Self-monitoring of blood glucose	□ Educate patients of diabetes management in the collaborative clinic. The knowledge will be printed out and given to patients for home study
Medication guidance	□ The importance of medication adherence □ Administration methods(oral & injection medication) □ Administration timing □ The injection technique of insulin □ Drug-drug interaction □ Drug storage	□ Create a WeChat group with all the enrolled patients in, and pharmacists will share diabetes knowledge periodically
Lifestyle intervention	□ Exercise □ Diet	□ Educate patients in the collaborative clinic □ Help patients make a healthy lifestyle plan
ADR * & complications identification and treatment	□ Recognition and prevention of hypoglycemia □ Identification and processing of complications of diabetes □ Identification and processing of ADR	□ Educate patients in the collaborative clinic □ Call the pharmacist if an emergency event occurs

*PS*: *ADR* Adverse drug reaction

#### Randomization and allocation concealment

The randomization protocol was used to select six out of 18 primary hospitals in Hunan province to carry out the study by using a standard protocol available in the website (http://stattrek.com/statistics/random-number-generator.aspx). There will be three centers in each group (intervention group and control group).

### Intervention protocol

#### Physician-pharmacist collaborative diabetes clinics

Three physician-pharmacist collaborative diabetes clinics will be established after the pharmacists get comprehensive training. Pharmacists in the collaborative clinics will provide several services which mainly consist of four aspects, including diabetes education, medication guidance, lifestyle intervention, and identification and treatment of adverse drug reaction and complications, as shown in Table 2.

Three measures will be taken to make sure that the project will be carried out smoothly in the collaborative clinics. 1) In the first week, a pharmacist from the Second Xiangya Hospital will stay in the collaborative clinic, monitor the trained local pharmacist and data collector’s work, and give him/her guidance to run the collaborative clinic well. 2) A pharmacist from the Second Xiangya Hospital will periodically go to the primary hospitals and check the progress of the project. Every week, there will be an internet meeting consisting of the PI, main participants and the pharmacists from the intervention group to report the process of the project, describe the problems they meet and exchange their experience in the collaborative clinics. 3) Data collectors are required to entry and upload the electronic CRF forms to the Second Xiangya Hospital within two days when they have finished the printed forms. A separate research staff will carefully check the data and immediately inform the data collectors to amend the errors once occur.

#### Usual care clinics

Similar to physicians in the collaborative clinics, physicians in the usual care clinics will provide routine services, mainly including diagnosis and treatment without education due to limited time. No pharmacists will be involved in the control group. Data collectors will present in the clinic to enroll patients and collect data of the assessments the same as in the collaborative clinics.

In order to ensure the implementation of the project in the usual care clinics, three measures will be taken: 1) In the first week, a pharmacist from the Second Xiangya Hospital will stay in the clinic and monitor the data collector’s work. A participant from the Second Xiangya Hospital will periodically go to the primary hospitals and check the progress of the project. 2) Every two weeks, an internet meeting consisting of the PI, main participants and the data collectors will be held to monitor the process of the project. 3) Same as the intervention group, data collectors are required to entry and upload the electronic CRF forms to the Second Xiangya Hospital within two days.

#### Follow-up visits and data collection

All the patients in either the intervention group or in the control group are required to visit their corresponding clinic at the 3rd, 6th, 9th and 12th month. They are allowed to arrive at the clinic within seven days before or after the appointed day. The measurement domains required to collect, survey methods and collection time-points are listed in Table 3.

**Table 3:** Measurement domains, survey methods and collection time-points

Variable	Component	Measurement methods	Baseline	3rd M	6th M	9th M	12th M
**Socio-demographic parameters**	Gender, age, telephone, address, education, occupation and annual income	Face-to-face interview	√				
**Clinical parameters**	Height, weight, waist to hip ratio, fasting blood glucose, 2-h postprandial blood glucose, HbA1c, diabetes complications, blood pressure and lipid files	Face-to-face interview, telephone or online interview	√	√	√	√	√
**Behavior parameters**	Medication adherence, diabetes’ knowledge, lifestyle, diabetes treatment target and treatment behavior		√ √	√	√	√	√ √
	Treatment satisfaction						√
**Economic parameters**	Direct costs (Average daily Medicine, outpatient clinic visit fees, and hospitalization fees if occurs), Indirect costs (deducted income due to absence of working, traffic and accommodation fees)	Face-to-face interview, telephone or online interview	√	√	√	√	√

In order to enhance patients’ follow-up rate, two measures will be taken: 1) The trained data collectors will send a text message to remind the patient three days before the appointed follow-up day. If the patient has not shown up in the clinic one day after the appointed follow-up day, they will call the patient. If the patient still insists not coming, the reasons will be recorded in the CRF form. 2) All the patients from either the control group or the intervention group will receive free tests of fasting blood glucose (FBS), 2-h postprandial blood glucose (2hPBG) and HbA1c at the 3rd, 6th, 9th and 12th month to sustain their involvement in the study.

### Outcomes

#### Primary outcomes measure

The primary outcome measure is glucose control (including fasting blood glucose, 2-h postprandial blood glucose and HbA1c) at 12 months’ follow-up adjusted for baseline value.

#### Secondary outcomes measures

Secondary outcomes are the following measures adjusted for baseline values:

1. patients’ medication adherence, diabetes knowledge and lifestyle;
2. patients’ hospitalization or emergency room visits and incidence of complications related to diabetes;
3. height, weight, waist to hip ratio, blood pressure and lipid files;
4. costs on diabetes management.

### Data monitoring

#### Monitoring of study process

The study will be divided into several tasks, and each task is scheduled to be finished in a certain time. The process of each task will be monitored and evaluated according to the timeline. Periodically, an internet meeting consisting of the PI, main research staff, data collectors will be held, and a main research staff from the Second Xiangya Hospital will go to the primary hospitals to check the progress of the project regularly.

#### Quality control procedures

Serial tests will be carried out during the 3 months’ training time to ensure the trained pharmacists are able to provide qualified services in the collaborative clinics in the primary hospitals. The trained data collectors will follow standard procedures to enroll patients, collect data and upload electronic data. An electronic CRF form of each patient is required to be uploaded to the Second Xiangya Hospital within two days. A separate staff who are in charge of data collection and analysis will check the data’s validity and integrity.

### Data analyses

The sample size was calculated by using the PowerSampleSize program (Vanderbilt University). The data will be analyzed using the SPSS software package (Statistical Package for the Social Sciences), version 22.0 for Windows (SPSS Inc., Chicago, Illinois, USA). Data will be checked for normal distribution by means of the Kolmogorov–Smirnov's test. Values are presented as mean ± standard deviation or median (range) for parametrically or nonparametrically distributed variables, respectively. In compliance with the distribution of data, Student's t-test or Mann–Whitney's U-test will be used for comparisons between the two groups. Chi-square or Fisher's exact tests will be used to compare between the groups with respect to categorical data. The follow-up assessment data will be analyzed using Generalized Linear Mixed Model and survival analysis. *P* < 0.05 is considered statistically significant. All tests are two tailed.

For cost-effectiveness analysis, we will collect the direct and indirect costs of each enrolled patient with T2DM, evaluate their quality of life through EQ-5D-3L utility value from data provided by patients, calculate quality-adjusted life years (QALYs), and analyze the effects of multiple variables by the Linear Mixed Effects Model. Based on this model, the differences between the average costs and the results will be analyzed during the 12 months. The incremental cost effectiveness (ICER) will be calculated, and the univariate and multivariate analyses will be performed.

### Sample size determination

Sample size is calculated using a two-tailed test and indicated that to detect an absolute difference of 0.76% in HbA1c in favor of the intervention group with α = 0.05, β = 0.1 (90% power), and standard deviation of 1.0%, a sample size of 36 patients per group was required [18]. An additional 10% was added to allow for patients dropping out of the study, making a target sample size of at least 40 patients in each primary hospital, same as a previous study [19]. Our target enrolled patient number in each hospital is 100, and we plan to complete patient recruitment in 6 months. If the eligible patients have reached 100 in less than 6 months, we will continue the enrollment until the end of the sixth month. If the enrolled patients have not reached our target number at the end of the sixth month, we will continue to enroll patients for an extra month and then stop. We estimate that the final included patient number is around 80 to 120 in each primary hospital.

### Covariates

The contents of the measures include four aspects, namely the socio-demographic parameters (age, gender, telephone, address, education, occupation and annual income), clinical parameters (Height, weight, waist to hip ratio, fasting blood glucose, 2-h postprandial blood glucose, HbA1c, diabetes complications, blood pressure and lipid files), process indicators (medication adherence, diabetes’ knowledge, lifestyle, diabetes treatment target and treatment behavior), and costs on diabetes.

### Dissemination

The research results will be disseminated through publications in peer-reviewed journals, academic conferences seminars and workshops. Articles and videos related to establishing physician-pharmacist collaborative diabetes clinics and diabetes management will be open to public in website for free.

## Discussion

It is urgent to establish feasible and cost-effective care mode to manage patients with diabetes in rural areas of China with the aim of ameliorating their glucose control, improving their medication adherence and lifestyle, and reducing the incidence of complications related to diabetes. There are two major reasons why people with T2DM in rural China have poor management. Firstly, physician density in rural areas is significantly lower than that in the urban areas. Due to lower income and limited professional development opportunities in the rural regions compared to the urban area, physicians in rural China are insufficient [20]. In 2013, physician densities at primary health centers and hospitals in rural areas were respectively 0.37 and 0.80 per 1,000 population, much lower than that in urban areas with 0.56 and 2.62 per 1,000 population [21–23]. Therefore, physicians in rural regions usually spend most of their time on disease diagnoses and treatment, while less time on patient education. Secondly, patients in rural China usually have lower socioeconomic status and education background, as a result, these patients are less aware of the importance of medication adherence, healthy lifestyle and regular monitoring of blood glucose levels [6]. Thus, patients in rural area often rush to the overloaded tertiary hospitals once they develop severe diabetic complications.

COVID-19 is the most infectious pandemic across the world in the past century, and the pandemic has placed unprecedented impact on health systems worldwide. Unfortunately, it is estimated that the pandemic may continue to exist for years. The Chinese government has taken several successful measures to prevent and control the pandemic, including aggressive containment strategy, hierarchical management, rational reallocation of resources and efficient contact tracing [24]. It is rational that many hospitals allocate a part of medical resources for COVID-19 prevention and control, resulting in reduced outpatient clinic capacities for chronic diseases care during the pandemic. However, it should be noted that insufficient glucose control will result in various acute and chronic complications, and the healthcare systems will have to cope with these after the COVID-19 pandemic. Moreover, diabetes has been proved as a susceptibility factor and independent predictor of worse prognosis in patients infected with SARS-CoV-2 [25, 26]. Therefore, it’s vital to improve diabetes management during the era of COVID-19 pandemic.

Studies have demonstrated that the pharmacist-physician collaborative care model are significantly helpful to improve the outcomes of patients with diabetes. A study consisting of 2,480 patients with diabetes included in both the collaborative care group and the usual care group from 6 hospitals and 22 patient-centered medical home practices showed that the pharmacist-physician collaborative care mode remarkably improved HbA1c, blood pressure and lipid files, as well as reduced hospitalizations compared to usual care, yielding an estimated cost savings of up to $2,619 per patient [27]. By providing the services of diabetes education and disease management via providing information and feedback to patients and recommendations to providers in the collaborative care practice, pharmacists helped more patients reached the goal of HbA1c below 7% [28]. Moreover, a systematic review consisting of 25 studies demonstrated that pharmacist-managed services in people with diabetes could save costs from $7 to $65,000 per person per year, and generated higher quality-adjusted life years [12]. Therefore, we should notice that in the era of COVID-19 pandemic, when part of physicians and nurses are taking responsibilities of preventing and controlling the pandemic, well trained pharmacists are valuable medical resources for diabetes management. The pharmacist-physician collaborative clinic may be a promising care model for patients with diabetes in China. Yet, to our knowledge, no study has ever investigated if this model could be adapted in primary hospitals of China. This proposed study will be the first to implement the pharmacist-physician collaborative clinic model to manage patients with T2DM in primary hospitals, and to evaluate the efficacy and cost-effectiveness of this program through a multi-site randomized controlled trial in Human province with 66.44 million residents. If effective, the physician-pharmacist collaborative clinics can be adapted and used in primary hospitals across China to improve glycemic control, enhance medication adherence, decrease incidence of complications and reduce patients’ dependence on physicians. Findings from the present study are meaningful for developing evidence-based diabetes care policy in rural China, especially in the COVID-19 pandemic era.

This proposed study has some limitations. Firstly, we will only carry out the study in Hunan province, where the economic level is at the upper middle level in China. The limited geographical reach of the proposed study may influence its generalizability. Secondly, we plan to follow up enrolled patients for only 12 months, the long-term effects of the physician-pharmacist collaborative clinics on the outcomes may be not known. We will follow up the patients if they prefer to continue staying in the collaborative clinics after one year intervention.

In summary, we will establish and evaluate the impacts of the physician-pharmacist collaborative clinics on diabetes management in primary hospitals of Hunan province in China. This project will be a great pilot to explore and show pharmacists’ new value in health care, and is expected to make a significant contribution to the development of a new evidence-based health policy for diabetes management, particularly for rural residents in China during and after the COVID-19 pandemic.

### Trial status

Recruitment has begun in May 2021.

## Data Availability

The datasets generated and/or analyzed during the current study are not publicly available but are available from the corresponding author on reasonable request.

## Abbreviations

T2DM: Type 2 diabetes mellitus
COVID-19: Coronavirus Disease 2019
WHO: World Health Organization
IDF: International Diabetes Federation
MTM: Medication therapy management
AE: Adverse event
PI: Principal Investigator
CRF: Case Report Form
FBS: Fasting blood glucose
2hPBG: 2-Hour postprandial blood glucose
SPSS: Statistical Package for the Social Sciences
QALYs: Quality-adjusted life-years
ICER: Incremental cost effectiveness ratio
